# Estimating the prevalence of sensitive behaviour and cheating with a dual design for direct questioning and randomized response

**DOI:** 10.1111/j.1467-9876.2010.00720.x

**Published:** 2010-08

**Authors:** Ardo van den Hout, Ulf Böckenholt, Peter G M van der Heijden

**Affiliations:** 1 Medical Research Council Biostatistics UnitCambridge, UK; 2 Northwestern UniversityEvanston, USA; 3 Utrecht UniversityThe Netherlands

**Keywords:** Bayesian inference, Cheating, Misclassification, Sensitive items, Social benefit fraud

## Abstract

Randomized response is a misclassification design to estimate the prevalence of sensitive behaviour. Respondents who do not follow the instructions of the design are considered to be cheating. A mixture model is proposed to estimate the prevalence of sensitive behaviour and cheating in the case of a dual sampling scheme with direct questioning and randomized response. The mixing weight is the probability of cheating, where cheating is modelled separately for direct questioning and randomized response. For Bayesian inference, Markov chain Monte Carlo sampling is applied to sample parameter values from the posterior. The model makes it possible to analyse dual sample scheme data in a unified way and to assess cheating for direct questions as well as for randomized response questions. The research is illustrated with randomized response data concerning violations of regulations for social benefit.

## 1. Introduction

When survey questions are asked about sensitive topics, respondents might be reluctant to provide a direct honest answer. To deal with this situation, Warner (1965) introduced randomized response (RR). This is an interview design where the observed answer to a question depends on the true status with respect to the topic as well as on a specified probability mechanism. The basic idea of RR is, first, that the probability mechanism protects the privacy of the respondent, and, second, that statistical inference is possible by incorporating the probability mechanism in the statistical model. Meta-analysis has shown that RR produces better prevalence estimates than other survey designs that deal with sensitive topics (Lensvelt-Mulders *et al.*, 2005).

The forced response design that was introduced by Boruch (1971) is an illustrative example of RR. In this design, a question is asked that requires a *yes* or a *no* as an answer. Instead of answering the question directly, the respondent throws two dice without revealing the sum of the dice. Next, the respondent follows a design: if the sum is 2, 3 or 4, the respondent answers yes. If the sum is 5, 6, 7, 8, 9 or 10, he answers the question truthfully. If the sum is 11 or 12, he answers no. Since the sum of the dice is hidden, the interviewer does not know whether the answer was forced by the design or provided truthfully. In this way, the privacy of the individual respondent is guaranteed.

The forced response design is a misclassification design. If we name the true status with respect to the question *latent*, then we can define and deduce conditional misclassification probabilities such as 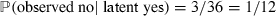, i.e. the probability of answering no in the RR design conditional on a true yes status. These probabilities are used to define the statistical model for the RR data.

It may not come as a surprise that some respondents do not follow the design when participating in an RR survey (Fox and Tracy, 1986; Clark and Desharnais, 1998; Böckenholt and Van der Heijden, 2007). We define *cheating* as the act of providing the least stigmatizing answer irrespectively of the outcome of the RR probability mechanism. In the forced response design, for example, answering no while the sum of the dice is 2 is cheating. There may be more than one reason for cheating. If a respondent does not understand the way that privacy is protected, he or she might be reluctant to co-operate. Likewise, general lack of trust with regard to the institute that conducts the survey may also induce cheating. Because of the way that RR works, cheaters cannot be identified. At the same time, it is clear that cheating causes extra perturbation that has to be taken into account in the model to obtain valid statistical inference.

Clark and Desharnais (1998) discussed cheating in a design with one RR question. They suggested using two samples where in each sample the same RR question is asked with different conditional misclassification probabilities. By combining the two samples, an RR model that takes cheating into account can be estimated. Inspired by the idea of using two samples, we propose a model to estimate cheating in a dual sampling scheme where a direct questioning (DQ) design and an RR design are applied to the same set of questions. The combination of the two designs provides information that is not obtainable by either design alone: we can estimate cheating simultaneously in both settings.

Our model is different from that in Clark and Desharnais (1998) since the latter is formulated for two differently specified RR designs with the assumption that the two designs induce the same level of cheating. Our model allows for different levels of cheating in the two designs. In addition, we relate the levels of cheating to individual covariates. The result is a general model that incorporates the model in Clark and Desharnais (1998) as a special case. Cruyff *et al.* (2007) investigated cheating by using a restricted log-linear model for RR data (no dual sampling scheme). The latter model can also been seen as a restricted version of our model.

In what follows, Section 2 describes the motivating data and Section 3 presents the models. In Section 4 identifiability is discussed and in Section 5 we compare our model with the model in Clark and Desharnais (1998). Section 6 analyses the data and Section 7 concludes the paper.

## 2. The data

Employees in the Netherlands are insured against loss of income caused by, for example, redundancy or disability. To receive social benefit, rules and regulations must be followed. The data that we consider are from the Social Welfare Survey that was conducted by the Dutch Department of Social Affairs in 2002. In this survey, individuals throughout the country were recruited for the sample if they had received disability benefit for at least 12 months before the study. We consider three related questions about possible rule violation. Question 1: have you recently done any small jobs for or via friends or acquaintances, for instance, in the past year or done any work for payments of any size without reporting it? (This pertains only to monetary payments.) Question 2: have you ever in the past 12 months had a job or worked for an employment agency in addition to your disability benefit without reporting it? Question 3: have you worked off the books in the past 12 months in addition to your disability benefit?

In the survey, 1760 individuals were asked these questions by using RR, and 467 individuals were asked these questions by using DQ. This choice of sample sizes yields similar efficiency regarding point estimates in the case of one RR question. In the RR sample, the same binary RR design was used across the three questions. The design is an adapted forced response design parameterized by 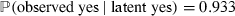 and 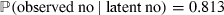; see Van den Hout and Lensvelt-Mulders (2005) for details. Denoting answers yes and no by 1 and 2 respectively, observed frequencies in the RR sample for the profile order 111, 112, 121, 122, 211, 212, 221 and 222 are given by 60, 48, 116, 269, 41, 144, 174 and 908. So, in the sample of 1760 individuals, 60 answered yes to all three questions, 48 answered yes to the first two questions, but no to the third, etc. For the DQ sample, these frequencies are 1, 1, 13, 24, 2, 2, 4 and 420.

In addition to the questions on rule compliance, information was collected on gender and age, and on attitude towards the rules of social benefit. We consider in this paper the attitude towards statement 1, ‘The rules are very reasonable’, and statement 2, ‘Not following the rules can be advantageous for me’. Answer categories are *completely agree*, *agree*, *neither agree nor disagree or do not know*, *disagree* and *completely disagree*.

## 3. Methods

This section starts with the standard RR model and the self-protective (SP) no model as introduced by Böckenholt and Van der Heijden (2007). Section 3.3 presents the model for the dual sample scheme.

## 3.1. Standard randomized response model

Let latent answers be denoted by *X* and observed answers by X^*^. The sample space for both stochastic variables is {1,…,*K*}. The general form of the RR designs in this paper is 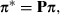 where 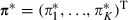 is a vector denoting the probabilities of observed answers, **π**=(π_1_,…,π_*K*_)^T^ is the vector of the probabilities of true (latent) answers and **P** is a specified non-singular *K*×*K* matrix of conditional misclassification probabilities 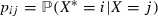. Matrix **P** will be called the *RR matrix*. Estimating **π**, i.e. the prevalence of the sensitive behaviour, is the aim of RR.

The RR matrix is 2×2 when the sensitive question is dichotomous. When questions are asked with more than two possible answers, we have *K*>2. It is also possible to combine RR questions which will result in *K*>2. For example, combining three binary RR questions yields *K*=8 answer profiles. The RR matrix for these eight profiles is **P**=**P**_1_⊗**P**_2_⊗**P**_3_, where ‘⊗’ is the Kronecker product and **P**_1_, **P**_2_ and **P**_3_ are the RR matrices of the three questions. This definition of **P** is based on the conditional independence assumption 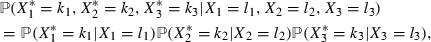 where *k*_1_,*k*_2_,*k*_3_,*l*_1_,*l*_2_,*l*_3_ ∈ {1,2}.

Assume that the sampling distribution is multinomial with parameters **π** and *n*. The vector with the observed frequencies is denoted 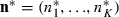. The likelihood function is given proportionally by 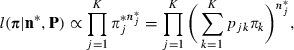
(1) where π_*k*_≥0 for *k* ∈ {1,…,*K*}, and 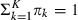.

For Bayesian analysis, we must specify a prior density. Given a multinomial sampling distribution, a possible choice of the prior density of **π** is the conjugate Dirichlet density with parameter **α**=(α_1_,…,α_*K*_), i.e. 
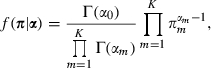
(2) where α_*m*_>0, for *m*=1,2,…,*K*, 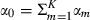 and Γ(·) denotes the gamma function (Gelman *et al.*, 2004). Parameters α_*m*_ can be interpreted as prior sample sizes for the *K* answer categories. A vague prior is defined as α_*m*_→0, for all *m*.

Combining expressions (2) and (1), the posterior density is given by 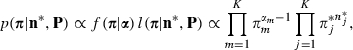 where π_*k*_≥0 for *k* ∈ {1,…,*K*}, and 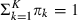 (Unnikrishnan and Kunte, 1999; Van den Hout and Klugkist, 2009).

## 3.2. Self-protective no model

Böckenholt and Van der Heijden (2007) presented an extended RR model that allows for the modelling of cheating. The assumption is that, if a respondent cheats, he or she always answers the least stigmatizing category. Typically, if the categories are yes and no, the cheater will always answer no. This behaviour is called SP no saying. By formulating the RR model in such a way that the least stigmatizing category is category *K*, the SP no RR model is given by 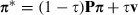
(3) where **v** is the *K*×1 vector with the *K*th entry equal to 1 and 0 elsewhere. Mixing weight τ is the cheating parameter and its interpretation is the percentage of the sample that cheats.

Identification can be an issue in the SP no model. We shall call an identifiability problem *intrinsic* when there are more parameters to estimate than there are independent observations. This is a special case of the more general problem of identifiability where two sets of parameter values correspond to the same probability density function (Casella and Berger, 2002). Without restriction on the parameters, model (3) has an intrinsic identifiability problem. Identifiability will be discussed in Section 4.

Define 
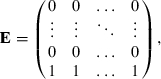
 with dimension *K*×*K*. Let **P**_τ_=(1−τ)**P**+τ**E**. The SP no model can be formulated as a misclassification model by π^*^=**P**_τ_**π**.

## 3.3. Model for dual sampling scheme

We specify an extended RR model that encompasses the above models, deals with the dual sampling scheme and relates individual covariates to the probability of cheating. The mixture model for respondent *i* in sample *s*=1,2 is given by 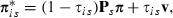
(4) where the RR design in sample *s* is given by **P**_*s*_. A logistic regression model is used to relate the probability of cheating to covariates, i.e. 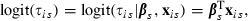
(5) where **β**_*s*_=(β_*s*.0_,β_*s*.1_,…,β_*s*.*p*_)^T^ and **x**_*is*_=(1,*x*_*is*.1_,…,*x*_*is*.*p*_)^T^. A log-linear model is specified for the latent distribution **π**, i.e. 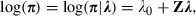 where **Z** is the model matrix. Parameter λ_0_ is not a free parameter but is derived from **Z** and **λ** to ensure that **π** is a valid probability vector with elements summing to 1. This will be illustrated in the application. Using a log-linear model makes it possible to test for association patterns in case **π** is defined with respect to a cross-classification. Cruyff *et al.* (2007) also used a log-linear model to analyse RR data while accounting for SP no. Their log-linear model was a restricted model to prevent an intrinsic identifiability problem. An advantage of the log-linear model is that maximization over possible values of **λ** is unrestricted whereas maximization over the independent elements in **π** is restricted to (0,1)^*K*−1^.

Assuming independence between the samples, the overall likelihood is the product of the likelihood for sample 1 and the likelihood for sample 2. Data consist of individual covariate values and indicators 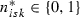, *i*=1,…,*n*_*s*_, where *s*=1,2 denotes the sample, *n*_*s*_ is the number of respondents in sample *s* and *k*=1,…,*K* is the category. Suppressing the conditioning on **P**_1_ and **P**_2_, the posterior density is given by 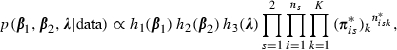 where 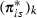 denotes the *k*th entry of vector 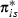 and *h*_1_(**β**_1_), *h*_2_(**β**_2_) and *h*_3_(**λ**) are the prior densities for **β**_1_, **β**_2_ and **λ** respectively.

Regarding computation, we follow the recommended procedure for mixture models and use unobserved indicators (Gelman *et al.* (2004), chapter 18). The mixture model (4) with mixing weight τ_*is*_ is viewed hierarchically; the observed 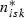 are modelled conditionally on unobserved indicators *c*_*is*_ for cheating, where *c*_*is*_=0 means that respondent *i* in sample *s* followed the RR instructions and *c*_*is*_=1 denotes cheating. The unobserved *c*_*is*_ are viewed as missing data and the parameter **β**_*s*_ is thought of as a hyperparameter determining the distribution of *c*_*is*_. The posterior density is thus given by 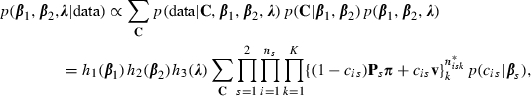 where the summation is over **C**={*c*_*is*_|*i*=1,…,*n*_*j*_,*s*=1,2} denoting the set with all possible realizations of the indicator variables. Note that *p*(*c*_*is*_|**β**_*s*_) is the logistic regression model that is defined by equation (5).

Sampling from the posterior can be undertaken by using the automatic Markov chain Monte Carlo functionality of WinBUGS (Lunn *et al.*, 2000). In the MCMC sampling, indicators are sampled from their distribution and model parameters are estimated conditionally on sampled indicators.

To show that the extended model can still be seen as a misclassification model, define for the dual sample scheme 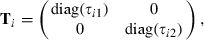 where diag(τ_*i*1_) is a *K*×*K* diagonal matrix with τ_*i*1_ on the diagonal and diag(τ_*i*2_) is defined likewise. Next define 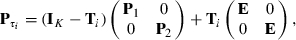 where **τ**_*i*_=(τ_*i*1_,τ_*i*2_)^T^, **I**_*K*_ is the 2*K*×2*K* identity matrix and **E** is defined as above. For 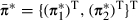 and 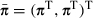, the dual sample SP no model for respondent *i* can be formulated as 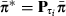.

Note that by specifying model (4) for only one sample, i.e. *s*=1, and using the intercept-only model for τ_*i*1_ and a log-linear model for **π** we obtain the SP no model in Cruyff *et al.* (2007). By further assuming that τ_*i*1_=0 and using the saturated log-linear model for *g*(**π**) we obtain the standard RR model.

## 4. Identification

Identification can be a problem in the SP no model as was discussed and illustrated in Van den Hout and Klugkist (2009), section 4.3. Given proper prior distributions, sampling from the posterior is possible with an unidentified model, but inference may be hampered or impossible owing to impractically wide credible intervals. For the situation where three binary RR questions were combined and *K*=8, Cruyff *et al.* (2007) dealt with the intrinsic identifiability problem in the SP no model (3) by specifying a log-linear model for **π** and restricting the three-way interaction to 0. In model (4) there is no intrinsic identification problem. Consider the situation in a dual sample scheme where three binary RR questions are combined and *K*=8. If we do not use individually observed covariate values and we refrain from further modelling of **π**, then the model is given by 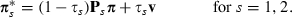
(6) 14 independent frequencies are observed: *K*−1=7 in the sample with the direct questions and seven in the RR sample. We must estimate nine independent parameters: π_1_,…,π_7_, τ_1_ and τ_2_. There is also no intrinsic identification problem when model (6) is defined for two binary RR questions (six independent frequencies; five independent parameters).

Even if there is no intrinsic identification problem, parameters might still be difficult to identify. Typically this will lead to high posterior correlation between the parameters and associated slow convergence of Markov chain Monte Carlo algorithms (Carlin and Louis (2009), page 153).

## 5. Comparison with the model of Clark and Desharnais (1998)

The model in Clark and Desharnais (1998) will be called the *CD model*. It can be seen as a dual sample SP no model. In this section, we briefly discuss this relationship.

Let us change the original parameterization π,β and γ in the CD model into α,β and γ respectively. Then α+β+γ=1. Parameter α is the proportion of honest yes respondents in the population, β is the proportion of honest no respondents and γ is the proportion of cheaters. The RR matrix in sample *s* for *s*=1,2 is given by 
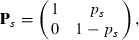
 where 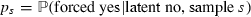. In this design, a latent yes always results in an observed yes. Privacy protection is induced by generating forced yes answers. There are no forced no answers.

The SP no formulation of the CD model in sample *s* for *s*=1,2 is given by 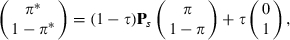 where 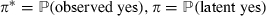 and τ is the cheating parameter.

The connection between the SP no and the CD model is given by α=(1−τ)π, β=(1−τ)×(1−π) and γ=τ. This shows that the CD model is a restricted version of model (4). There are no covariates and, more importantly, in the CD model the probability of cheating is assumed to be the same in both samples.

The SP no formulation of the CD model illustrates possible ways of reporting results. When estimated α, β and γ in the CD model are reported, the estimated prevalence is given for the proportions in the population of honest yes respondents, honest no respondents and cheaters, without further assumptions regarding the group of cheaters. Another possibility is to report the estimated π as the prevalence of the sensitive category in the population. This implies, however, assuming that the cheaters are a random sample from that population (with prevalence given by the estimated τ).

## 6. Data analysis

Let sample 1 denote the RR questions and sample 2 the direct questions. This dual sample scheme has three binary sensitive questions. The prevalence for the 2^3^=8 profiles is given by **π**=(π_1_,π_2_,…,π_8_)^T^ which we shall write as **π**=(π_111_,π_112_,…π_222_)^T^ to represent the eight profiles 111, 112, …, 222 better. The model with all the covariates is given by 
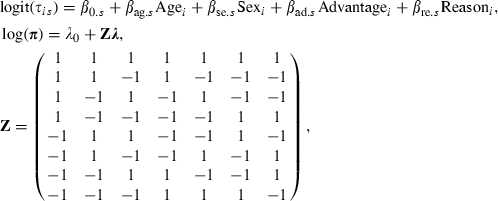
 where **λ**=(λ_1_,…,λ_7_)^T^ and 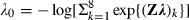, with (**Zλ**)_*k*_ being the *k* entry of vector **Zλ**.

In the logistic regression models we used centred age by using Age = observed age −50 years. Covariate Sex is coded 0 for women and 1 for men. The scale for the attitude towards statement 1 and statement 2 is centred by using zero as an understandable reference point. The coding reflects an assumed scale on compliance. For Advantage, categories are from −2 for completely disagree up to 2 for completely agree. For Reason, categories are from −2 for completely agree up to 2 for completely disagree. In this way, higher scores on these covariates may be related to increased non-compliance with the rules. Table 1 shows the distribution of the answers to the statements. Overall the distributions are similar across the designs. The largest difference is for Reason for category 1 (disagree).

**Table 1:** Distribution of the answers to the two statements in the RR design and in the DQ design

Design	Results for statement 1: Reason					Results for statement 2: Advantage				
	−2	−1	0	1	2	−2	−1	0	1	2
RR	0.18	0.46	0.25	0.08	0.03	0.18	0.35	0.25	0.16	0.06
DQ	0.12	0.44	0.24	0.15	0.05	0.16	0.36	0.31	0.12	0.05

## 6.1. Inference

We did a preliminary analysis by maximum likelihood estimation. First, we estimated the model without covariates (model I; nine parameters) given by equation (6) and used the prevalence estimates as starting values in the maximization for the model with all the covariates (model III; 17 parameters) as given above. Next, we selected those covariates in model III that were significant regarding the univariate Wald test with significance level 10%: Sex and Advantage for sample 1; Advantage for sample 2. This restricted version of model III is called model II and it has 12 parameters. Model I can be fitted to observed frequencies. Owing to the sample-specific SP no parameters τ_1_ and τ_2_, model I induces a perfect fit for the frequencies for category *K*=8 (the 222-category) in both the RR sample and the DQ sample.

To compare the models, we use the Bayesian information criterion (BIC) that provides a rough approximation to the Bayes factor that is independent of the priors (Carlin and Louis (2009), page 53). Information criteria are given in Table 2. In accordance with the theory, minus twice the log-likelihood decreases when the number of parameter increases. The BIC takes the increase of parameters into account and shows a clear preference for model II. Following the BIC, we shall discuss Bayesian inference for model I and model II.

**Table 2:** Information criteria for the maximum likelihood estimation: minus two times the log-likelihood −2LL and the BIC

Model	Number of parameters	−2LL	BIC
I	9	5872.2	5941.6
II	12	5824.8	5917.3
III	17	5810.0	5941.1

For the priors we choose vague uniform priors, i.e. for the individual λs and βs we specify a uniform distribution on the interval (−10,10). For example, for β_se.1_ a value 10 would mean that the odds on cheating increase multiplicatively by exp (10) when men are compared with women. This is rather unlikely. We call the prior that is specified by the interval (−10,10) vague because the interval is sufficiently wide to include all realistically possible values of the parameters without favouring specific values. The same reasoning applies to parameter λ_7_ which is the three-factor interaction and describes how the odds ratio between two variables changes across categories of the third. For example, define ϑ_11.*c*_ as the odds ratio for questions 1 and 2 given category *c* of question 3; we have ϑ_11.1_/ϑ_11.2_= exp (8λ_7_) and a value of ±10 for λ_7_ is quite extreme.

The models are mixture models and long Markov chain Monte Carlo chains are recommended. We used a burn-in of 50 000 simulations and 50 000 updates. Convergence was checked by assessing the chain visually, by looking at the auto-correlation, and by Geweke's convergence diagnostic (Geweke, 1992) as implemented in the R package (Plummer *et al.*, 2006). Given the Bayesian framework, transformations from λs to πs or from βs to τs are direct and credible intervals (CIs) are readily derived. Results for model I and model II are presented in Table 3.

**Table 3:** Bayesian inference for models without covariates (model I) and with covariates (model II)

Parameter	Results for model I	Results for model II
		
	Posterior mean (95% CI)	Posterior mean (95% CI)
π_111_	0.017 (0.006, 0.029)	0.017 (0.006, 0.029)
π_112_	0.002 (0.0001, 0.008)	0.002 (0.0001, 0.008)
π_121_	0.058 (0.040, 0.078)	0.059 (0.040, 0.079)
π_122_	0.115 (0.079, 0.157)	0.120 (0.086, 0.158)
π_211_	0.006 (0.001, 0.015)	0.006 (0.001, 0.019)
π_212_	0.006 (0.001, 0.018)	0.007 (0.001, 0.019)
π_221_	0.019 (0.005, 0.043)	0.021 (0.006, 0.044)
π_222_	0.776 (0.710, 0.831)	0.768 (0.707, 0.819)
τ_1_	0.157 (0.097, 0.218)	
τ_2_	0.536 (0.330, 0.694)	
β_0.1_ (intercept)		−1.899 (−2.787,−1.271)
β_se.1_, Sex		−0.968 (−1.976,−0.276)
β_ad.1_, Advantage		−0.845 (−1.358,−0.469)
β_0.2_ (intercept)		−0.292 (−1.480,0.531)
β_ad.2_, Advantage		−1.370 (−2.220,−0.727)

First we discuss model I. This is the model without the covariates and it can be assessed on the level of the frequencies. As a consequence, it is easy to investigate the goodness of fit by posterior predictive checking (Gelman *et al.* (2004), section 6.2). Denote the 16 observed frequencies (eight in sample 1; eight in sample 2) generically by n^*^, and the model parameter vector by **θ**. The Pearson χ^2^-statistic for observed frequencies n^*^ and estimated frequencies derived from **θ** yields a posterior predictive *p*-value that is equal to 0.085. This shows that the model fits the data though the evidence is not overwhelming and further modelling seems worthwhile.

For the estimated model I, note that the posterior mean of τ_1_ is smaller than the posterior mean of τ_2_ and that the CIs do not overlap. This means that the probability of cheating in sample 1 with RR is smaller than the cheating in sample 2 with DQ. This is in accordance with the basic idea of RR. When sensitive questions are asked, a technique that protects the privacy of respondents leads to improved compliance with the design of the survey.

Next we discuss model II, which we consider to be the final model. Monitoring the mean of the individually estimated cheating probabilities yields posterior means 0.155 for sample 1 and 0.568 for sample 2, which are close to the posterior means of the cheating parameters for model I given by 0.157 and 0.536 respectively. Furthermore, according to the estimation of β_ad.1_ and β_ad.2_, when an individual states that it is not advantageous to violate a benefit rule, he or she is more likely to cheat in the survey (the posterior means of β_ad.1_ and β_ad.2_ are negative and the CIs do not include zero). The posterior distribution of β_se.1_ shows that men are less likely to cheat in the RR sample than women.

Given that often not following the rules can indeed be advantageous, we think that the attitude question is a sensitive question. Individuals who do not follow the RR rules also do not honestly answer the attitude question. In other words, denying that violating the benefit rules can be advantageous is a proxy for cheating in the RR design.

**Fig. 1:**
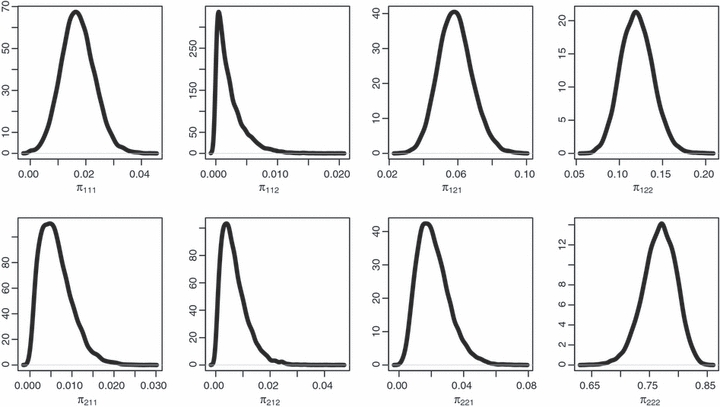
Posterior densities of prevalence parameters estimated by using the model with the covariates

The prevalence estimates are also given in Table 3. Comparing the posterior means for **π**=(π_111_,…,π_222_)^T^ the results show that the prevalence estimates are robust regarding model selection. The probability of complying with all the benefit regulations is 0.768 with 95% CI (0.707, 0.819), whereas the probability of violating all the regulations is 0.017 (0.006, 0.029). It is interesting to see that there is a relatively large probability that individuals violate the first regulation but follow the second and the third: 0.120 (95% CI (0.086, 0.158)). Fig. 1 brings out the strength of the Bayesian framework: the asymmetrical distributions of some of the prevalence parameters is nicely captured by the MCMC results. Distributions that are not close to the boundary of the parameter space (for instance, those of π_111_ and π_121_) resemble the shape of normal distributions.

## 6.2. Sensitivity analysis

We investigated the sensitivity of the results with respect to the specification of the prior. When changing the specification of the prior for the individual λs and βs to a uniform distribution on the interval (−100,100), inference is very similar to the values that are reported in Table 3—except for β_se.1_. For this parameter the median of the simulated values is still close to the value that is reported in Table 3, but the mean is −5.660, which is caused by simulating some large negative values. This means that β_se.1_ is only weakly identified in the model. Changing the specification of the prior to a uniform distribution on the interval (−5,5) gives results that are very close to those in Table 3. For β_se.1_ we obtain a posterior mean −0.9285 and 95% CI (−1.84,−0.2787). Overall we conclude that the inference is robust regarding the specification of the vague priors. The exception is inference for β_se.1_—which is a parameter which is only weakly identified. It is still reasonable to state that men are less likely to cheat in the RR sample than women, i.e. β_se.1_<0, but better to refrain from further quantification of this effect.

Model II can also be formulated regarding the combination of two questions. This specifies three models: one for questions 1 and 2, one for questions 1 and 3, and one for questions 2 and 3. For these three models, the parameters are π_11_,π_12_,π_21_,π_22_,β_0.1_,β_se.1_, β_ad.1_,β_0.2_ and β_ad.2_. Of course, the interpretation of these parameters varies according to the model at hand. We think that combining all the three questions provides the best information regarding prevalence and cheating behaviour, but assessing the models for the combination of just two questions provides some insight into the sensitivity regarding the estimation of cheating. Consider the mean of the individually estimated probabilities of cheating for sample 1 and 2. As already mentioned, for the combination of all three questions, the posterior means and 95% CIs of the mean of the individual cheating parameters are given by 0.155 (0.077, 0.218) and 0.568 (0.400, 0.670). For questions 1 and 2, we obtain 0.194 (0.098, 0.294) and 0.608 (0.431, 0.747), for questions 1 and 3, 0.198 (0.114, 0.301) and 0.635 (0.481, 0.765), and, for questions 2 and 3, 0.190 (0.097, 0.294) and 0.5825 (0.329, 0.789). Although there are some deviations, overall, estimating cheating seems quite robust across the different models. Note also that not one of the CIs overlaps when comparing cheating in the RR design with cheating in the DQ design. As was to be expected, the bivariate models are less accurate than the model for the three questions taken together—this was of course the main reason for choosing the latter for investigating prevalence and cheating.

## 7. Discussion

To estimate prevalence and cheating, we have used a model for a dual sample scheme with questions about compliance with social benefit rules. The questions were asked by using RR in one sample and the same questions were asked directly (DQ) in the second sample. The combination of DQ and RR provides information that is not obtainable by either method alone: we can estimate cheating for DQ, and—because the model is identified—we can avoid making assumptions about higher order interaction effects in the RR model. The model can also be used in surveys with two different RR designs, where the cheating is allowed to vary across the designs. This is important as it is often not realistic to assume that two different RR designs induce similar cheating behaviour.

Cruyff *et al.* (2007) used maximum likelihood estimation to analyse data from a series of RR questions. Cheating was investigated by using a model for SP no saying, where the identifiability problem was dealt with by restricting a log-linear model for latent probabilities. The SP no model was estimated with one parameter. Clark and Desharnais (1998) also used maximum likelihood for data that were obtained with two RR designs for the same question. Their model can be seen as a dual model with SP no, but cheating in both designs is assumed to be the same and is estimated with one parameter. Assuming different cheating behaviour within this model would lead to an identifiability problem. Van den Hout and Klugkist (2009) used Bayesian inference for data that were obtained from a series of RR questions. Ways of cheating (among which is SP no) were investigated without posing restrictions on latent probabilities. Instead, the identifiability problem was dealt with by a stepwise procedure with regard to the minimum level of cheating that induces model fit. The SP no model was estimated with one parameter. The dual model that is presented in the current paper extends previous RR models and makes new and important inference possible. By formulating a model for RR and DQ, and by using data from a series of questions, the model allows us to distinguish and investigate cheating behaviour in DQ and RR.

The reason for cheating is often complex and will vary across RR surveys. Many factors may be involved, e.g. the sensitivity of the question, potential repercussions if the true status is disclosed, the understanding of the protection of privacy, the people who administer the questions and the number of questions. If the dual design consists of two RR designs (instead of an RR design and a DQ design), the detection of cheating may be related to the difference in parameterization of the designs. A bigger difference makes the estimation of cheating more efficient. However, a bigger difference also means that one design may protect significantly less than the other, in which case the two designs may induce different cheating behaviour.

It is possible to formulate a model for a dual sample scheme in the case with one RR question, but such a model will often be difficult to estimate. To give an example, assume that we want to estimate cheating parameters 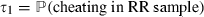 and 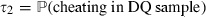. To identify the model, we split each sample according to gender. To justify the split, we must assume that gender does not interact with the probability of cheating. (This assumption, however, is not supported by the inference in this paper.) The model for rule violation is now defined by logit(π_women_)=θ_0_ and logit(π_men_)=θ_0_+θ_1_. In this way we estimate the four parameters τ_1_, τ_2_, θ_0_ and θ_1_ given four independently observed frequencies: yes answers for men and yes answers for women in sample 1, and yes answers for men and yes answers for women in sample 2. For the data at hand, this model for one RR question could not be estimated as the simulated values from the posterior were not identified on the support that is specified by vague priors.

It is advantageous to investigate cheating in an RR design with more than one question. For that reason, we analysed our data with regard to the cross-classification of three questions and used the cross-classifications of only two questions for a sensitivity analysis. When there is more than one RR question, there is more information on cheating, which—when taken into account—will lead to a better estimation of the prevalence of the behaviour of interest.

A Bayesian framework was used (for other Bayesian RR analyses, see, for example Winkler and Franklin (1979), Unnikrishnan and Kunte (1999), Fox (2005) and Van den Hout and Klugkist (2009)). A frequentist approach would work as well as illustrated by the preliminary maximum likelihood analyses in the application (see also the frequentist log-linear RR models analysis in Cruyff *et al.* (2007)). However, there are a few advantages of the Bayesian framework. Bayesian CIs are more appropriate than frequentist asymptotic confidence intervals in the case of parameter estimates near the boundary of the parameter space. The importance of this was illustrated in the application. In general, boundary solutions are very common in the analysis of RR data as the sensitive issues that are investigated are often linked to behaviour that has a low prevalence in the population. With the freely available software WinBUGS, the Bayesian framework has gained another advantage: it is now relatively easy to implement and investigate RR models. As an example of possible prior information, assume that a standard RR design for a binary variable is applied every year with the same question. If in all the previous years the estimated prevalence was below 5%, Bayesian inference with an informative prior seems reasonable. We might for instance choose **α**=(α_1_,α_2_) with α_1_=1 and α_2_>1 for the Dirichlet density.

Although specific RR data motivated this research, the paper presents a general approach that allows for various choices of RR designs. We have noted quite a number of references where RR is compared with DQ; see, for example, the surveys that were discussed in the meta-analysis by Lensvelt-Mulders *et al.* (2005). If there is some suspicion of cheating, then the data from those comparisons can be reanalysed by using our model.

As mentioned above, cheating in RR designs is complex and we do not want to pretend that our model for the dual sample scheme captures all that is at stake. Further research is needed and it would be interesting to see how the model (or variants thereof) would work with other data sets. That being said, we think that our model is the best description of the data that is currently available. The difference in estimated cheating behaviour concurs with research showing that RR induces more co-operation from respondents than DQ. By combining the two samples, we gain efficiency with respect to the estimation of both the prevalence and the cheating. Relating the probability of cheating to covariate values seems reasonable. The sensitivity analysis that considers two questions at a time shows the robustness of the analysis: when analysing the variables two by two, we obtain similar results.
